# Brain Functional Network in Chronic Asymptomatic Carotid Artery Stenosis and Occlusion: Changes and Compensation

**DOI:** 10.1155/2020/9345602

**Published:** 2020-09-23

**Authors:** Shihao He, Ziqi Liu, Zongsheng Xu, Ran Duan, Li Yuan, Chu Xiao, Zhe Yi, Rong Wang

**Affiliations:** ^1^Department of Neurosurgery, Beijing Tiantan Hospital, Capital Medical University, Beijing 100070, China; ^2^Department of Neurosurgery, Peking University International Hospital, Beijing 102206, China; ^3^State Key Laboratory of Cognitive Neuroscience and Learning & IDG/McGovern Institute for Brain Research, Beijing Normal University, Beijing 100875, China; ^4^Jishuitan Hospital, Fourth Clinical College of Peking University, Beijing 100096, China; ^5^Center of Stroke, Beijing Institute for Brain Disorders, Beijing 10069, China

## Abstract

Asymptomatic carotid artery stenosis (CAS) and occlusion (CAO) disrupt cerebral hemodynamics. There are few studies on the brain network changes and compensation associated with the progression from chronic CAS to CAO. In the current study, our goal is to improve the understanding of the specific abnormalities and compensatory phenomena associated with the functional connection in patients with CAS and CAO. In this prospective study, 27 patients with CAO, 29 patients with CAS, and 15 healthy controls matched for age, sex, education, handedness, and risk factors underwent neuropsychological testing and resting-state functional magnetic resonance (rs-fMRI) imaging simultaneously; graph theoretical analysis of brain networks was performed to determine the relationship between changes in brain network connectivity and the progression from internal CAS to CAO. The global properties of the brain network assortativity (*p* = 0.002), hierarchy (*p* = 0.002), network efficiency (*p* = 0.011), and small-worldness (*p* = 0.009) were significantly more abnormal in the CAS group than in the control and CAO groups. In patients with CAS and CAO, the nodal efficiency of key nodes in multiple brain regions decreased, while the affected hemisphere lost many key functional connections. In this study, we found that patients with CAS showed grade reconstruction, invalid connections, and other phenomena that impaired the efficiency of information transmission in the brain network. A compensatory functional connection in the contralateral cerebral hemisphere of patients with CAS and CAO may be an important mechanism that maintains clinical asymptomatic performance. This study not only reveals the compensation mechanism of cerebral hemisphere ischemia but also validates previous explanations for brain function connectivity, which can help provide interventions in advance and reduce the impairment of higher brain functions. This trial is registered with Clinical Trial Registration-URL http://www.chictr.org.cn and Unique identifier ChiCTR1900023610.

## 1. Introduction

Asymptomatic carotid artery stenosis (CAS) and carotid artery occlusion (CAO) are characterized by the presence of extracranial internal carotid atherosclerotic stenosis in the ipsilateral carotid perfusion region in individuals without a recent history of ischemic stroke or transient ischemic attack (TIA) [1, 2]. Stroke is often an important factor leading to impaired neurocognitive function in patients [3–6]. Severe internal carotid artery (ICA) stenosis (>50%) is associated with a higher incidence rate of silent cerebral infarction [7]. Notably, studies have demonstrated that mice with unilateral CAO show significant object recognition disorder, despite maintaining normal spontaneous activity [8, 9]. These findings indicate that “asymptomatic” stenosis and occlusion may not be asymptomatic.

Functional neuroimaging can reveal brain activity, and thus, it has become an important tool in the study of neurological diseases [10]. Changes in functional connectivity in neurological and psychiatric diseases, including Alzheimer's disease (AD), epilepsy, schizophrenia, traumatic brain injury, multiple sclerosis, and coma, have provided pathological perspectives on the global and local indices of brain networks [11], and these perspectives can be used to explain some of the cognitive deficits associated with these diseases [12, 13]. Although cerebral perfusion insufficiency or infarction may be the cause of disease progression of CAS and CAO, the mechanisms underlying subsequent changes in brain function (such as cognitive decline) [14–16] and network connectivity have not been clarified [3, 15, 17–19]. However, the progressive changes in the cerebral network in patients with CAS and CAO and the compensation of cerebral network in the affected cerebral hemisphere remain to be clarified. Studying how the brain compensates in an ischemic environment not only reveals functional changes in the disease state but also validates previous annotations of brain function. This information is important because it helps provide interventions in advance and reduce the impairment of higher brain functions.

The purpose of this study was to determine the relationship between changes in brain network connectivity and the progression from internal CAS to CAO through graphical theoretical analysis. Therefore, we used resting-state functional magnetic resonance imaging (rs-fMRI) to compare brain network connections in 27 patients with CAO, 29 patients with CAS, and 15 healthy controls (HCs).

## 2. Materials and Methods

### 2.1. Subjects and Inclusion and Exclusion Criteria

This prospective study enrolled 27 patients with CAO and 29 patients with CAS from the Neurosurgery Department of Beijing Tiantan Hospital affiliated to Capital Medical University between March 2019 and December 2019.

The inclusion criteria were as follows: (1) findings of digital subtraction angiography (DSA) or carotid ultrasound examination which were consistent with the diagnostic criteria of CAS and CAO [20, 21]; for the CAS group, ≥70% stenosis on the affected side of the carotid artery and <50% stenosis of the contralateral carotid artery; (2) for the CAO group, complete occlusion of the affected internal carotid artery for more than 4 weeks and <50% stenosis of the contralateral carotid artery; (3) right hand dominance; (4) absence of a history of stroke, dementia, or major psychiatric disease; and (5) a minimum education level of primary school

The exclusion criteria were as follows: (1) presence of posterior circulation diseases; (2) other causes of carotid stenosis including chronic inflammatory arteritis which were ruled out; (3) presence of other neuropsychiatric diseases and severe systemic diseases (e.g., Alzheimer's disease, Parkinson's disease, and history of stroke); (4) the manifestations of any medications that could affect the cognitive function; and (5) any contraindications for MR scan (e.g., metal implants). We also recruited 15 HCs whose age, sex, and education level were similar to those of the patient groups as possible.

### 2.2. Ethical Statements

Written informed consent was obtained from all participants. This study was conducted in accordance with the principles of the Declaration of Helsinki and was approved by the Institutional Review Board of Beijing Tiantan Hospital, Capital Medical University (KYSQ2019-058-01).

### 2.3. MRI Acquisition

MRI data were obtained using a 3.0-Tesla MR system (Verio A Tim+Dot System, Siemens, Germany). A standard 12-channel head coil (3T Head MATRIX, A Tim Coil, Siemens) was used for signal reception. Each subject lay supine with the head snugly secured by a belt and foam pads. In rs-fMRI scans, subjects were asked to close their eyes, not to fall asleep, and not to think about anything in particular. The scanning parameters were as follows: repetition time (TR), 2220 ms; echo time (TE), 30.0 ms; voxel size, 3.0 × 3.0 × 3.0 mm; field-of-view (FOV), 192 mm; slice thickness, 3.0 mm; number of slices, 32; and total scanning time, 9 min and 11 sec.

### 2.4. Data Preprocessing

rs-fMRI data preprocessing was conducted using SPM 12 (Wellcome Department of Imaging Neuroscience, London, UK; https://www.fil.ion.ucl.ac.uk/spm/software/spm12/) implemented in MATLAB (Matlab Release 2013b, Mathworks Inc., Natick, MA). The first six volumes of individual functional images were discarded to achieve magnetization equilibrium. Slice-timing correction was implemented to align rs-fMRI images according to the middle slice. Subsequently, individual images were realigned (the standard of removal: 3 mm), so that each part of the brain is in the same position on every volume and warped into the standard MNI space by applying the transformation matrix that can be derived by registering the T1 image (coregistered with functional images) into the MNI template by using unified segmentation. Smoothing (4 × 4 × 4 mm) was used to improve the signal-to-noise ratio and to attenuate anatomical variances caused by inaccurate inter-subject registration after spatial normalization. Nuisance signals were removed from each voxel's time series to reduce the effects of nonneuronal fluctuations, including head motion profiles and cerebrospinal fluid (CSF) and WM signals. rs-fMRI data were bandpass-filtered to reduce the effects of low-frequency drift and high-frequency physiological noise. Regions of interest (ROIs) were placed using the Anatomical Automatic Labeling (AAL) atlas. Pearson's correlations for all time-course pairs were computed for each participant and transformed into *z*-scores via Fisher's transformation.

### 2.5. Functional Network Analysis

Graph theory analysis were performed according to the following steps implemented in GRETNA software [22]. Preprocessed rs-fMRI images were structurally defined into the AAL-90 atlas. GRETNA contains parcellation schemes defined by randomly parcelling the brain into 1024 ROIs. The mean time series was extracted from each parcellation unit, and pairwise functional connectivities were estimated among the time series by calculating linear Pearson's correlation coefficients. After calculating Pearson's correlation coefficient (*r*) in each ROI pair, a 45 × 45 hemispheric correlation matrix was constructed for each subject. For network analysis, various topological properties of a network were calculated using both global and nodal characteristics, which can be compared with random network counterparts to determine nonrandomness.

For graph theory analysis, six node-based and three global parameters were obtained for each network. The node-based network parameters included nodal-clustering coefficient (*C*), shortest path length, nodal efficiency, nodal local efficiency (E loc), degree centrality (DC), and betweenness centrality (BC); the global parameters included global efficiency (E glob), small-worldness (*s*), assortativity (*A*), hierarchy, and network efficiency. Finally, we calculated the area under the curve (AUC) for each network metric. Mathematical definitions of these parameters have been described elsewhere [23].

We used ICA to preprocess data using the Group ICA of fMRI toolbox (GIFT 4.0a, http://icatb.sourceforge.net/), which runs an Infomax algorithm. The preprocessed group data were decomposed into 43 spatial independent components (ICs). The data were concatenated and reduced using two-stage principal component analysis (PCA) and ICs and then calculated using the Infomax algorithm. The GICA-3 back-reconstruction step was used to separate single-subject components from the set of aggregate components calculated in the previous step. Finally, for all subjects, the acquired spatial component maps were converted into *z*-score maps. The brain networks were divided based on their anatomical and functional properties and included default-mode network (DMN), primary visual network (Prim_visual), higher visual network (High_visual), left executive control network (LECN), and right executive control network (RECN).

### 2.6. Statistical Analysis

One-way analysis of variance (ANOVA) was performed to compare continuous variables. Contingency tables of Pearson's *χ*^2^ test and Fisher exact test were used to compare categorical variables between three groups. Differences were considered statistically significant when *p* values were <0.05. Statistical analyses were performed using SPSS software, version 20 (IBM Corp, Armonk, NY). In the rs-fMRI data network analysis, patients were divided into three groups and bilateral cerebral hemispheres were differentiated; repeated measure analysis of variance (rmANOVA) was used with Bonferroni or FDR correction [24].

## 3. Results

Clinical variables of the patients with right internal carotid atherosclerosis and those of HCs are shown in Table 1. There were no statistically significant differences in sex, education level, and risk factors, such as hypertension and diabetes, between these groups. To ensure further accuracy of the analysis, we selected 15 normal subjects to be included in the HC group after a rigorous examination of the MRI data. There was no statistically significant difference in age between the three groups, that is, the left CAO and CAS groups and the control group. However, age was slightly different between the right CAO and CAS groups; therefore, when we processed the imaging data, the age of the right CAO and CAS groups was treated as a covariable.

Among the network attributes of the left CAO and CAS groups, the global attributes were not significantly different. However, the positive results of node indices are mostly consistent with the differences between the left and right hemispheres. In addition, there were only a few significant differences in edge attributes. Therefore, the results of the brain network analysis in the left CAO and CAS groups and their clinical basis variables are provided as supplementary material (Supplementary Table 1).

### 3.1. Alterations in Global Network Properties

**Figure 1:**
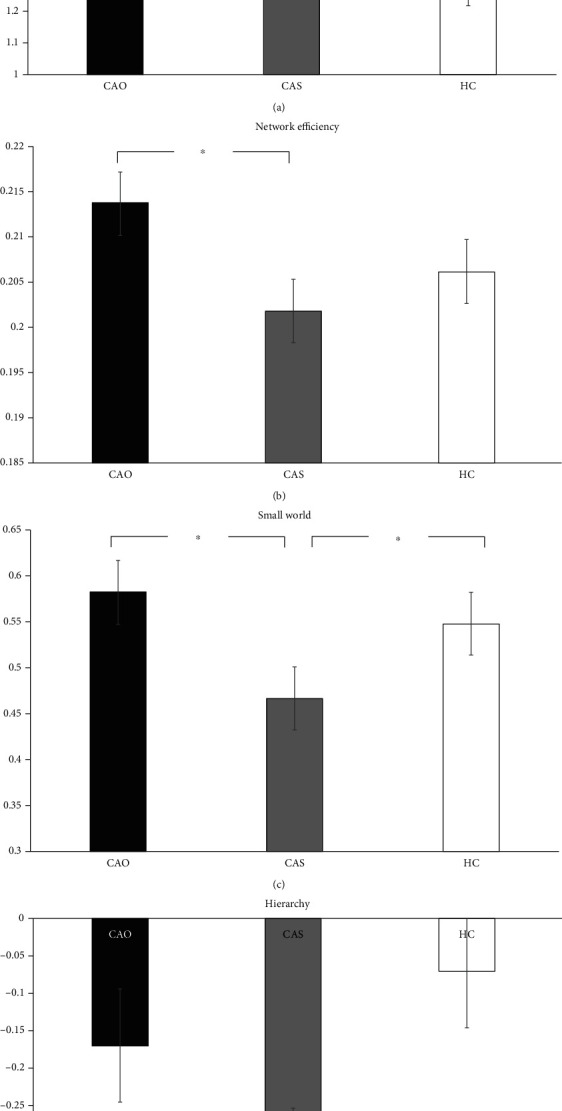
Graphical representation of four global attributes. ^∗^*p* < 0.05.

Figure 1 shows four global network properties with statistically significant differences. Assortativity—a main effect of subjects—was significant (*F*(2, 42) = 7.487, *p* = 0.002), and the index value of the HC group was significantly lower than those of the CAO and CAS groups. Hierarchy—a main effect of subjects—was significant (*F*(2, 42) = 7.181, *p* = 0.002), and the value of the CAS group was significantly lower than that of the HC group. The results of these two indicators were the first to be found in chronic ischemic brain disease. Network efficiency—a main effect of subjects—was significant (*F*(2, 42) = 4.998, *p* = 0.011), and the value of the CAO group was significantly higher than that of the CAS group. Small-worldness—the main effect of subjects—was significant (*F*(2, 42) = 5.276, *p* = 0.009), and the value of the CAS group was significantly lower than those of the CAO and HC groups.

### 3.2. Alterations in Regional Nodal Characteristics

**Figure 2:**
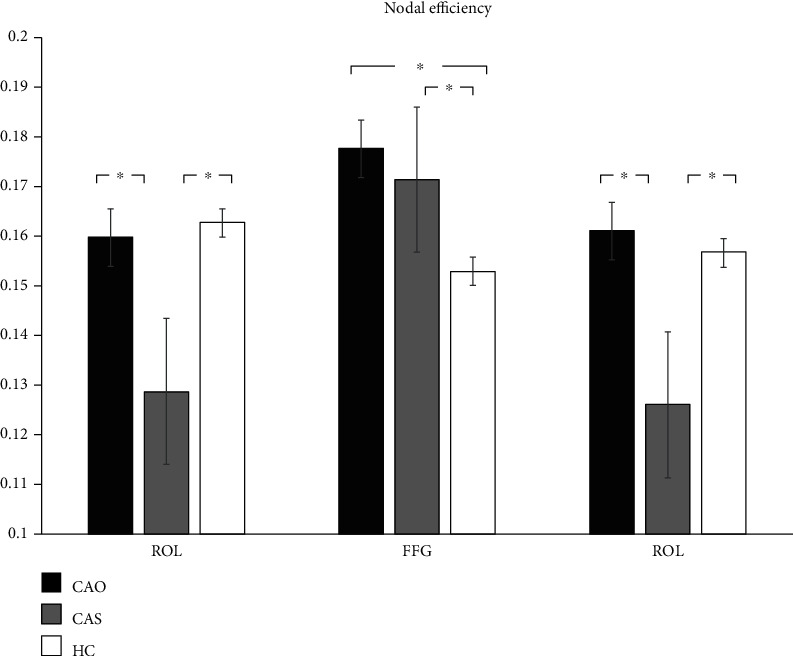
Graphical representation of node differences. ROL: Rolandic operculum; FFG: fusiform gyrus; TPOmid: temporal pole, middle temporal gyrus. The 45 nodes were subjected to repeated measurement ANOVA of 3 × 2 and corrected by FDR, and the *p* value was set at 0.05.

The 6 node-based network parameters—*C*, shortest path length, nodal efficiency, E loc, DC, and BC—were averaged across hemispheres and compared between the ipsilateral and contralateral sides of stenosis and occlusion in the patients and between the left and right sides in the controls. In the 3 groups, with regard to square difference analysis, there are some differences in the nodes between the two hemispheres; however, because one of the hemispheres in the brain itself is the dominant hemisphere, we focused on the statistical differences among the three groups. As shown in Figure 2, there are statistically significant differences among the three nodes with regard to nodal efficiency attributes, after correction. Node 28 FFG is smaller in the HC group than in the CAO and CAS groups. Nodes 9 ROL and 44 TPOmid are smaller in the CAS groups than in the CAO and HC group.

### 3.3. Alterations in Functional Connectivity

**Figure 3:**
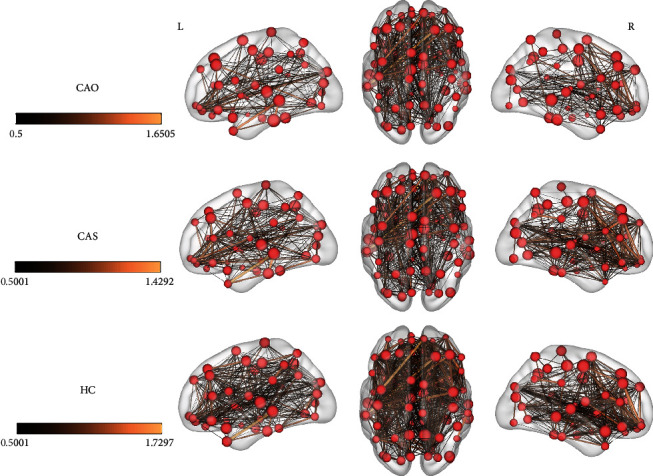
The connectivity maps of three groups (*r*‐threshold = 0.5, density = 0.25).

Figure 3 illustrates the typical brain networks derived from rs-fMRI of two patients. Sparser functional connectivity was observed in the hemisphere of the stenotic side in these patients.

**Figure 4:**
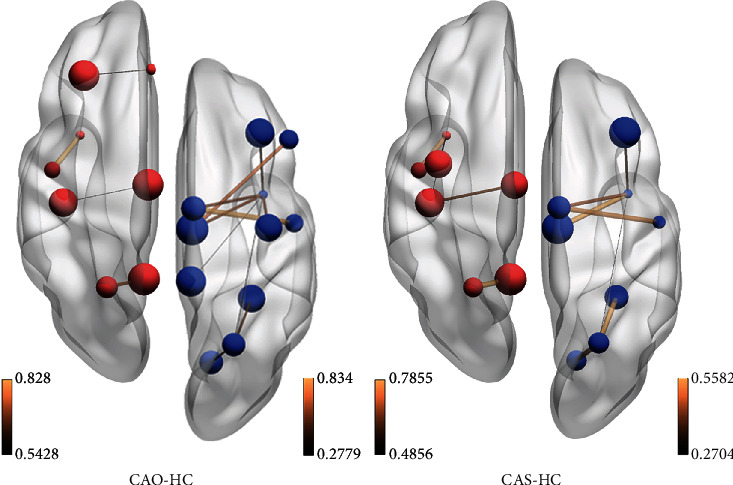
Map of differences in brain functional connections. In the figure on the left, interactions between brain regions in the left hemisphere are higher in the CAO group than in the HC group (red node), and those in the right hemisphere are lower in the CAO group than in the HC group (blue node). In the figure on the right, interactions between brain regions in the left hemisphere are higher in the CAS group than in the HC group (red node), and those in the right hemisphere are lower in the CAS group than in the HC group (blue node).

*p* was set at 0.001 in the first step and at 0.05 in the second step, and significant differences in edge attributes were found.  Figure 4 shows statistically significant differences between types of subjects and interactions between brain regions (*p* = 0.0030).

In the right cerebral hemisphere of patients with CAO and CAS of the right carotid artery, connections between the Rolandic operculum and supplementary motor area, middle frontal gyrus and insula, supplementary motor area and insula, insula and median cingulate and paracingulate gyri, insula and superior parietal gyrus, lingual gyrus and superior parietal gyrus, and fusiform gyrus and superior parietal gyrus were reduced. However, three connections, that is, connections between the precentral gyrus and insula, inferior frontal gyrus, triangular part with median cingulate and paracingulate gyri, and insula and inferior temporal gyrus, that reduced in the CAO group were more significant than those in the CAS group.

In the left cerebral hemisphere of patients with CAO and CAS of the right carotid artery, connections between the Rolandic operculum and insula, median cingulate and paracingulate gyri and postcentral gyrus, and superior parietal gyrus and precuneus were stronger than those in normal controls. However, two connections, that is, connections between the precentral gyrus and insula and between insula and postcentral gyrus that appeared in the CAS group, were more significant than those that appeared in the CAO group. The connection between the middle frontal gyrus and anterior cingulate and paracingulate gyri is even more significant in the CAO group than in the CAS group.

**Figure 5:**
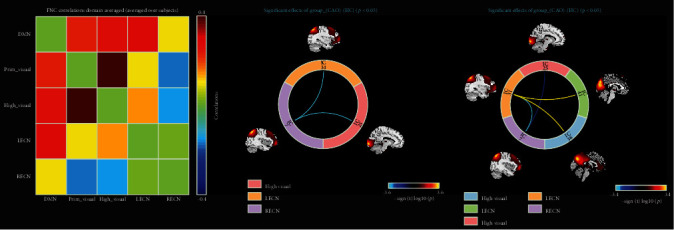
Map of differences in functional network connectivity.

We show the results of FNC analysis in Figure 5. Compared with HC, the connections between the right executive control network and the other two networks, the LECN and the higher visual network, were significantly decreased in the CAO patients. More disorders were found in CAS patients, in which the functional connection between the left and right executive control network decreased. And there was a significant decrease in the connection between the RECN and the higher visual network. At the same time, we found an increase in the connection between the LECN and two other functional networks, the DMN and the primary visual network.

## 4. Discussion

This rs-fMRI-based prospective study is the first study to fully elucidate the similarities and differences and compensatory connections in the brain between patients with asymptomatic CAS and CAO. Our findings suggest that changes in brain network connectivity indicators are more sensitive to hemispheric detection. Our results show that the global attributes of the patient's brain network and the efficiency of nodes in multiple brain regions decreased, while the affected hemisphere lost many key functional connections among patients with CAS and CAO.

Because of the effect of the dominant hemisphere, this study focuses more on the main effects of groups than on the differences between the left and right sides of the brain. We included the results of differences between the brain regions in the supplementary material. (Supplementary Figure G-H).

In the analysis of the global network parameters, no significant differences were observed between the controls and patients. Only when data on each hemisphere were processed independently (by using 45 × 45 correlation matrix), a significant difference was found in the global efficiency of the controls and patients [25]. Therefore, when we analyzed the global network properties, the overall analysis may not have reflected the subtle changes in connectivity resulting from insufficient blood supply by one carotid artery, and the analysis of the affected hemisphere may be more accurate. We hypothesized that functional connectivity in areas affected by hemodynamics, but not globally, was indeed impaired in patients with carotid stenosis. Deterioration in hemodynamics not only increases the risk of ischemic events but also alters the brain activity.

In patients with affected right carotid artery, there were significant differences in four global attribute indices (Figure 1). First of all, both the CAS and CAO groups showed higher assortativity than did the HC group, a finding that was almost unknown in brain network studies of ischemic disease; and this change, which was found on the basis of our hemispheric analysis, may be a specific neuroimaging marker of the disease. Assortativity is a measure of the strength (weighted degrees) of correlation between connected nodes [26], and it reflects the tendency of the nodes to be connected to other nodes of the same or similar strength. It ranges between −1 and 1. If a network has an assortative attribute of degree, it means that the nodes with high assortativity in the network tend to be connected to those with high assortativity, and nodes with low assortativity tend to be associated with those with low assortativity [27]. In a study of gliomas, contralesional assortativity was found to be associated with scores of the complex attention and cognitive flexibility domain [28]. This is characteristic of an assortative network. A negative assortativity value implies that the hubs of the network are not connected to each other, which is characteristic of a disassortative network. An assortative network is thought to be resilient to disruption (e.g., removal of nodes), because the core of highly connected nodes provides redundancy and facilitates the spread of information over the network [27]. In the CAS and CAO groups, this coordination and the spread of information on the network were clearly impaired. In addition, the network efficiency of patients with CAS also decreased significantly. The efficiency in patients with CAO did not decrease; it is slightly higher than that in HCs. Effects of CAO may be compensated by the contralateral carotid artery and bilateral vertebral artery after the occurrence of a carotid artery blockage, and there may also be collateral circulation and other compensatory blood supplies; therefore, there is a slight increase in this indicator. However, we need a larger sample size to clarify whether these parameters really differ between the CAO and HC groups.

Networks that are cheap to build but still efficient in propagating information are called economic small-world networks. Small-worldness is an attractive model to characterize brain networks because the combination of high local clustering and short-path length supports the two fundamental organizational principles in the brain: functional segregation and functional integration [29]. With regard to the small-world attribute, we noted that it was significantly lower in the CAS group than in the CAO and HC groups. A small-worldness network represents high cluster and short-path length topology [30]. Small-worldness is a frequently used indicator of information transfer efficiency, and the decline observed in the CAS group is often associated with cognitive impairment in previous studies [25]. This phenomenon was not obvious in the CAO group, and instead, there was a slight increase. However, it did not mean that the CAO network was more efficient in transmitting information, and this may be related to other compensatory vessels or new collateral circulation.

We assume that in an organization's command communication network, if all the commands are one way from top to bottom, the whole network is considered very hierarchical; therefore, the number of two-way connections is small, and the hierarchy is closer to 1 (number of edges actually connected in both directions)/(number of edges that can be connected in both directions, i.e., *C*(*n*, 2)). Conversely, if in an organization A can give orders to B and B can give orders to A, then, the organization is not so hierarchical. Therefore, compared to the other two groups, the brain network of CAS patients is less hierarchical, and the network tends to be disordered.

Among the indicators of global attribute, we found several specific indicators of the cerebral ischemia group. In patients with CAS, brain networks are affected very severely, and the indicators related to the speed of network information transmission have declined significantly, while still showing a fluctuating and reconfigurable hierarchy phenomenon. However, in the chronic CAO group, although the unilateral blood flow was completely occluded, the performance of the brain network was significantly better than that of the CAS group because of the existence of collateral circulation compensation, which indicated the presence of a strong plasticity and self-mediation.

Besides, the indices of nodal efficiency were significantly different among the three groups with regard to different brain regions, namely, the Rolandic operculum, fusiform gyrus, and temporal pole (middle temporal gyrus). In terms of anatomical functions, the temporal pole and fusiform gyrus are often used as supplementary language functional areas outside Wernicke's area. Recent evidence indicates that this region is not critical for speech perception or for word comprehension. Rather, it supports retrieval of phonological information, which is used for speech output and short-term memory tasks [31]. Among the three groups, the CAS group showed the worst performance, especially in the middle temporal gyrus (temporal pole), where the decrease in nodal efficiency might cause corresponding dysfunction.

As Figure 3 shows, the more severe the disease, the lesser the connections are in the whole brain. Although we observed more disorganization in both global and local attributes in patients with CAS, fewer connections were found in patients with CAO, possibly because of complete disruption of blood flow in one cerebral hemisphere in this patient population.

There are several similarities between the results of functional connectivity analysis of the brain network in these two chronic ischemic diseases (Figure 4). This is generally consistent with our hypothesis that stenosis and occlusion are ischemic diseases that change slowly and have the same pathological process. The focus of this study was to explore the differences in the topological properties of patients with right-sided disease. Therefore, we first examined the right hemisphere of the brain in both the groups. The results clearly indicate that patients with CAO had a lower functional connectivity in the right hemisphere than did the normal control group. This result indicated that the function of the affected hemisphere is impaired in patients with CAO, that is, in those without the right carotid artery blood supply, even if other collateral circulation is established. It affects areas such as the frontal lobe, fusiform gyrus, insula, and cingulate gyrus. At the same time, the left hemisphere of the brain has a corresponding functional connection to compensate. In the FNC analysis, not surprisingly, CAO patients with disease in the right hemisphere showed a significant decrease in the connection between the RECN and the other two networks, the LECN and the higher visual network. The executive control network (ECN) and default mode network (DMN) belong to task-positive systems, and the visual network belongs to the sensor cortex systems. The executive control network includes the multiple medial prefrontal cortex and inferior frontal and inferior parietal regions, with the dorsolateral prefrontal cortex (dlPFC) at its core. These brain regions are mostly associated with inhibition of activity, mood, etc. The control network participates in many advanced cognitive tasks and plays an important role in adaptive cognitive control [32]. In CAS patients, the performance was the same as that of CAO patients, except that the connection of LECN to two other networks, DMN and primary visual network, was significantly increased. DMN has been linked to a wide range of neuropsychiatric disorders including Alzheimer's disease, autism, schizophrenia, and epilepsy. In particular, reduced activity in the default mode network was associated with autism, while hyperactivity was observed in schizophrenia. In Alzheimer's disease, amyloid deposition caused by the course of the disease causes the default mode network to be compromised in the first place [33]. From these examples, we can see the important role of DMN in brain activity. In the case of cerebral hemodynamics of CAS patients, the enhancement of DMN connections at this time reflects the strong plasticity of the brain.

Our results contribute to the understanding of normal brain function networks, explore changes in brain connectivity in asymptomatic patients with chronic ischemic encephalopathy, and identify potential compensation mechanisms for changes in brain hemodynamics.

Our study has some limitations. Brain networks are complex and diverse, and further studies with a large sample size are thus needed to determine possible differences in functional connectivity between patients and controls and to understand the effects of changes in blood circulation in CAS and CAO on the brain network and advanced nervous function. In addition, patients with different handedness and stenosis may show different types of cognitive impairments. Therefore, further studies including more patients with different handedness are needed to confirm our current findings.

## 5. Conclusions

In conclusion, a comparison of the differences among the three groups using graph theory analysis showed four indicators of abnormal cerebral network (assortativity, hierarchy, network efficiency, and small-worldness), which occur as a result of the disruption of hemodynamics in the brains of patients with CAS and CAO. Furthermore, the nodal efficiency of key nodes in multiple brain regions of patients with CAS and CAO decreased, while the affected hemisphere lost many key functional connections. In the FNC analysis, the decline of the connection between multiple functional networks was also found. However, partial compensation occurred in the contralateral cerebral hemisphere, which may be the reason for the clinical asymptomatic manifestations. And the increase of functional network connectivity between LECN and DMN and primary visual network reflects the strong plasticity of the brain. The above-mentioned results indicate a correlation between impaired functional connectivity and clinical higher neurological function before the occurrence of real clinical symptoms because of the existing functional connectivity impairment in the local brain regions in the asymptomatic state. Out future studies will be focused on the exploration of methods for predicting the condition, providing interventions in advance, and reducing the impairment of higher brain functions.

## Figures and Tables

**Table 1:** Basic characteristics of study participants.

	CAS (*n* = 16)	CAO (*n* = 15)	HC (*n* = 15)	*p* value
Age (years)	58.94 ± 5.32	55.53 ± 9.80	61.33 ± 8.226	0.145
Male : female	2.2	6.5	2.75	0.570
Education (years)	8.29 ± 2.26	9 ± 1.90	8.87 ± 2.95	0.795
Risk factors (%)				
Hypertension	9 (56.3)	7 (46.7)	7 (46.7)	0.871
Diabetes mellitus	4 (25)	5 (33.3)	5 (33.3)	0.851
Ischemic heart disease	2 (12.5)	1 (6.7)	2 (13.3)	1.000
Hypercholesterolemia	6 (37.5)	7 (46.7)	4 (26.7)	0.555
Smoking	7 (43.8)	8 (53.3)	6 (40)	0.812

Age and years of education are represented as the median and standard deviation. The risk factors are presented as the number of people and percentage. The chi-square test was used for the analyses.

## Data Availability

All data that support the findings of this study are available upon request from the corresponding author.

## Supplementary Materials

Supplementary Materials Table 1: basic characteristics of study participants (left). Alterations in regional nodal characteristics. Alterations in functional connectivity. Supplementary Figure A: CAO—the left hemisphere < the right hemisphere (5-9, 5-15, 6-19, 15-19, 9-21, 15-21, 4-22, 5-22, 15-22, 15-27, 15-38, 27-29, 20-40, 21-40, and 38-40). Supplementary Figure B: CAS—the left hemisphere > the right hemisphere (6-19, 15-19, 15-21, 4-22, 5-22, 15-22, 15-27, 9-33, 20-40, and 38-40). Supplementary Figure C: left hemisphere—CAO < HC (5-9, 6-19, 9-21, 15-21, 5-22, 27-29, and 38-40). Supplementary Figure D: left hemisphere—CAO < CAS (15-19 , 9-21, 15-21, 4-22, 5-22, 9-33, 27-29, 21-40, and 38-40). Supplementary Figure E: right hemisphere—CAO > CAS (15-28 and 20-40). Supplementary Figure F: right hemisphere—CAO > HC (20-40). Additional images of patients with right carotid stenosis and occlusion. Supplementary Figure G: CAO—the right hemisphere < the left hemisphere (9-10, 1-15, 4-15, 9-15, 10-15, 4-16, 7-17, 15-17, 15-29, 17-29, 15-30, 24-30, 28-30, 30-34, and 15-45). Supplementary Figure H: CAS—the right hemisphere < the left hemisphere (1-15, 4-15, 9-15, 10-15, 4-16, 7-17, 15-17, 15-29, 17-29, 15-30, 24-30, 28-30, 30-34, and 15-45).
