# Supplementary material for: Brain Functional Network in Chronic Asymptomatic Carotid Artery Stenosis and Occlusion: Changes and Compensation
Source: Neural Plast. 2020 Sep 23;2020:9345602. doi: 10.1155/2020/9345602 (PMC7530486; doi:10.1155/2020/9345602)
Supplement: Supplementary Materials — Table 1: basic characteristics of study participants (left). Alterations in regional nodal characteristics. Alterations in functional connectivity. Supplementary Figure A: CAO—the left hemisphere < the right hemisphere (5-9, 5-15, 6-19, 15-19, 9-21, 15-21, 4-22, 5-22, 15-22, 15-27, 15-38, 27-29, 20-40, 21-40, and 38-40). Supplementary Figure B: CAS—the left hemisphere > the right hemisphere (6-19, 15-19, 15-21, 4-22, 5-22, 15-22, 15-27, 9-33, 20-40, and 38-40). Supplementary Figure C: left hemisphere—CAO < HC (5-9, 6-19, 9-21, 15-21, 5-22, 27-29, and 38-40). Supplementary Figure D: left hemisphere—CAO < CAS (15-19 , 9-21, 15-21, 4-22, 5-22, 9-33, 27-29, 21-40, and 38-40). Supplementary Figure E: right hemisphere—CAO > CAS (15-28 and 20-40). Supplementary Figure F: right hemisphere—CAO > HC (20-40). Additional images of patients with right carotid stenosis and occlusion. Supplementary Figure G: CAO—the right hemisphere < the left hemisphere (9-10, 1-15, 4-15, 9-15, 10-15, 4-16, 7-17, 15-17, 15-29, 17-29, 15-30, 24-30, 28-30, 30-34, and 15-45). Supplementary Figure H: CAS—the right hemisphere < the left hemisphere (1-15, 4-15, 9-15, 10-15, 4-16, 7-17, 15-17, 15-29, 17-29, 15-30, 24-30, 28-30, 30-34, and 15-45). [file 9345602.f1.docx]

**Supplementary material**

**Supplementary Table 1**. **Basic Characteristics of Study Participants (Left)**

|  | CAS(n=13) | CAO(n=12) | HC(n=15) | p value |
| --- | --- | --- | --- | --- |
| Age(years) | 61±9.15 | 60.14±9.75 | 61.33±8.226 | 0.944 |
| Male：Female | 5.5 | 11 | 2.75 | 0.518 |
| Education(years) | 10.15±2.44 | 8.75±2.38 | 8.87±2.95 | 0.329 |
| Risk factors(%) |  |  |  |  |
| Hypertension | 7(53.8) | 5(41.7) | 7(46.7) | 0.922 |
| Diabetes mellitus | 4(30.8) | 4(33.3) | 5(33.3) | 1 |
| Ischemic heart disease | 4(30.8) | 3(25) | 2(13.3) | 0.573 |
| Hypercholesterolemia | 4(30.8) | 5(41.7) | 4(26.7) | 0.703 |
| Smoking | 6(46.2) | 5(41.7) | 6(40) | 1 |

**Alterations in Regional Nodal Characteristics**

We performed repeated measurement anova of 3×2 for 45 nodes, corrected by FDR, and p value card 0.05. Degree

No.12,13,14 The left hemisphere > the right hemisphere

No.20 The left hemisphere < the right hemisphere

NodalEfficiency

No.20 The left hemisphere < the right hemisphere

**Alterations in Functional Connectivity**

Supplementary Figure A

CAO: The left hemisphere < the right hemisphere 5-9、5-15、6-19、15-19、9-21、15-21、4-22、5-22、15-22、15-27、15-38、27-29、20-40、21-40、38-40


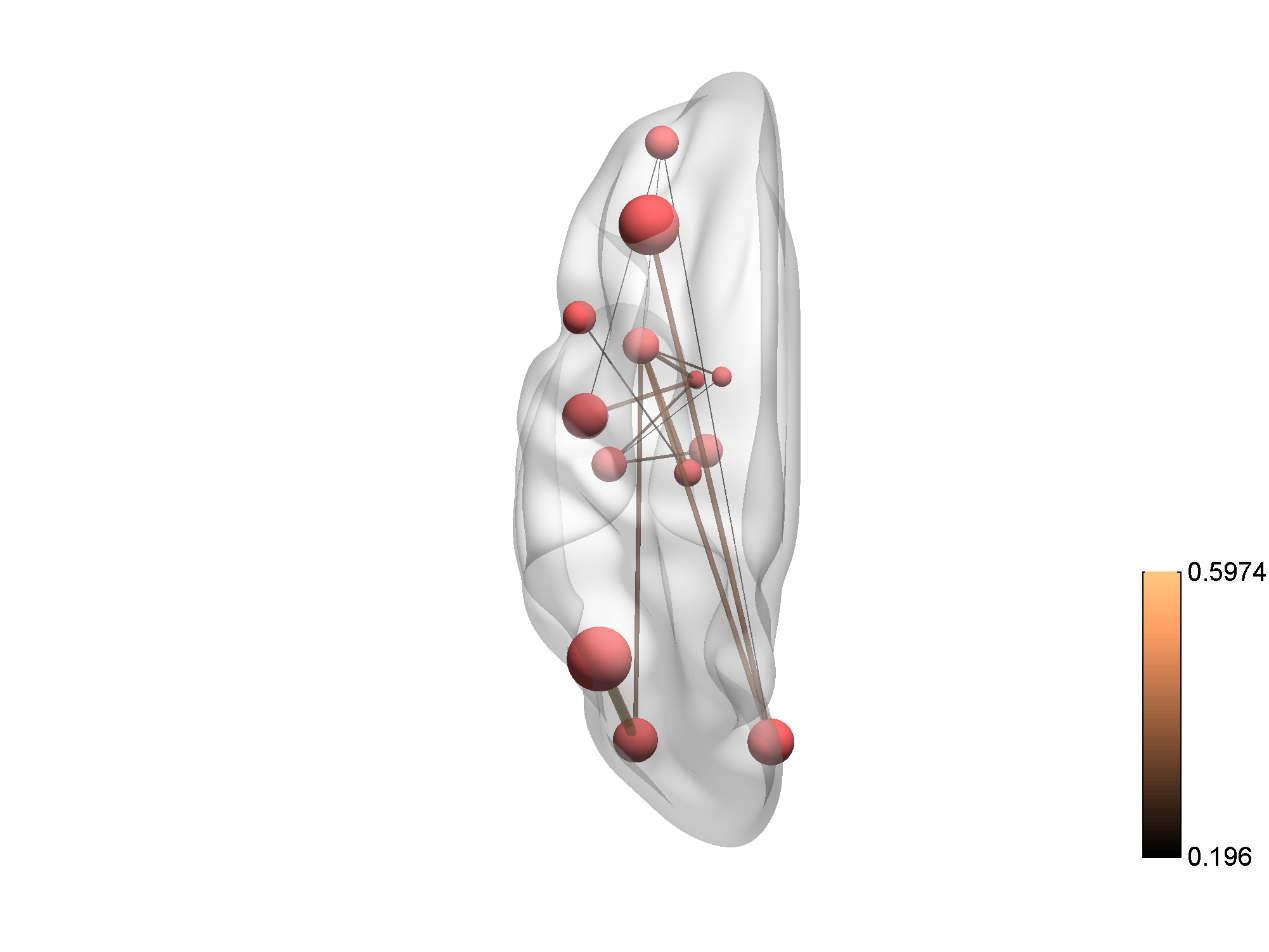


Supplementary Figure B

CAS: The left hemisphere > the right hemisphere 6-19、15-19、15-21、4-22、5-22、15-22、15-27、9-33、20-40、38-40


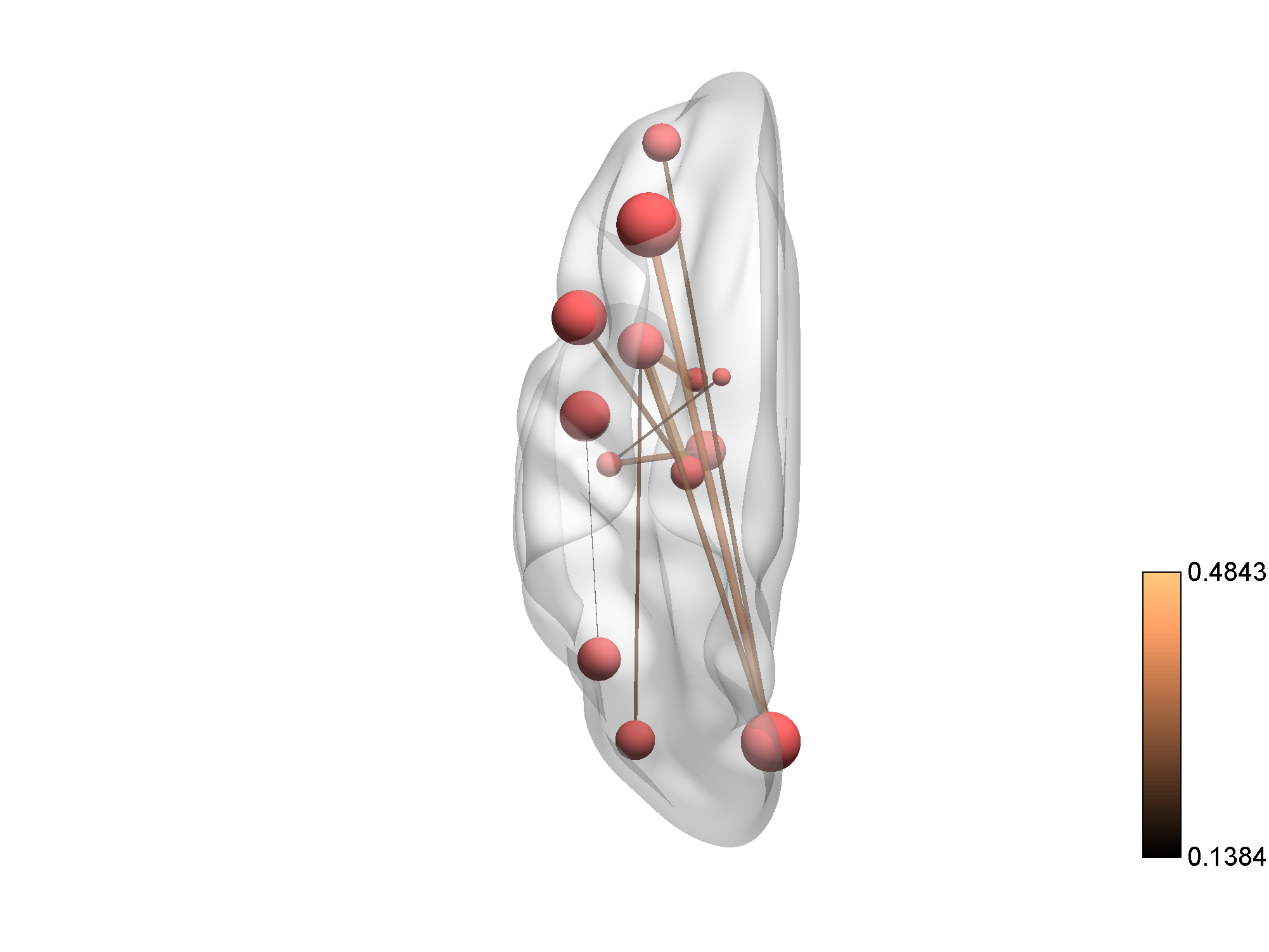


Supplementary Figure C

Left hemisphere : cao < hc 5-9、6-19、9-21、15-21、5-22、27-29、38-40


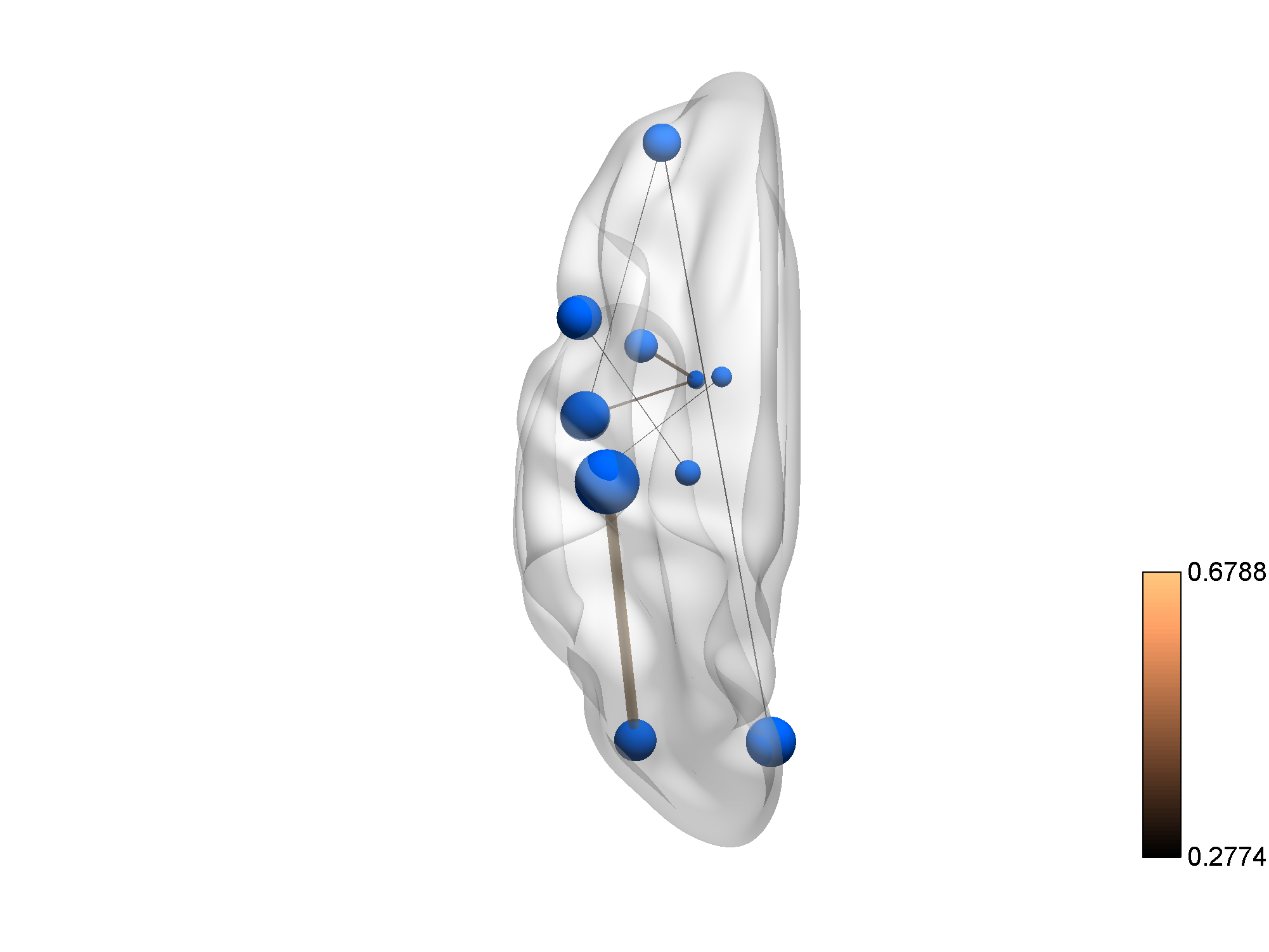


Supplementary Figure D

Left hemisphere：cao < cas 15-19 、9-21、15-21、4-22、5-22、9-33、27-29、21-40、38-40


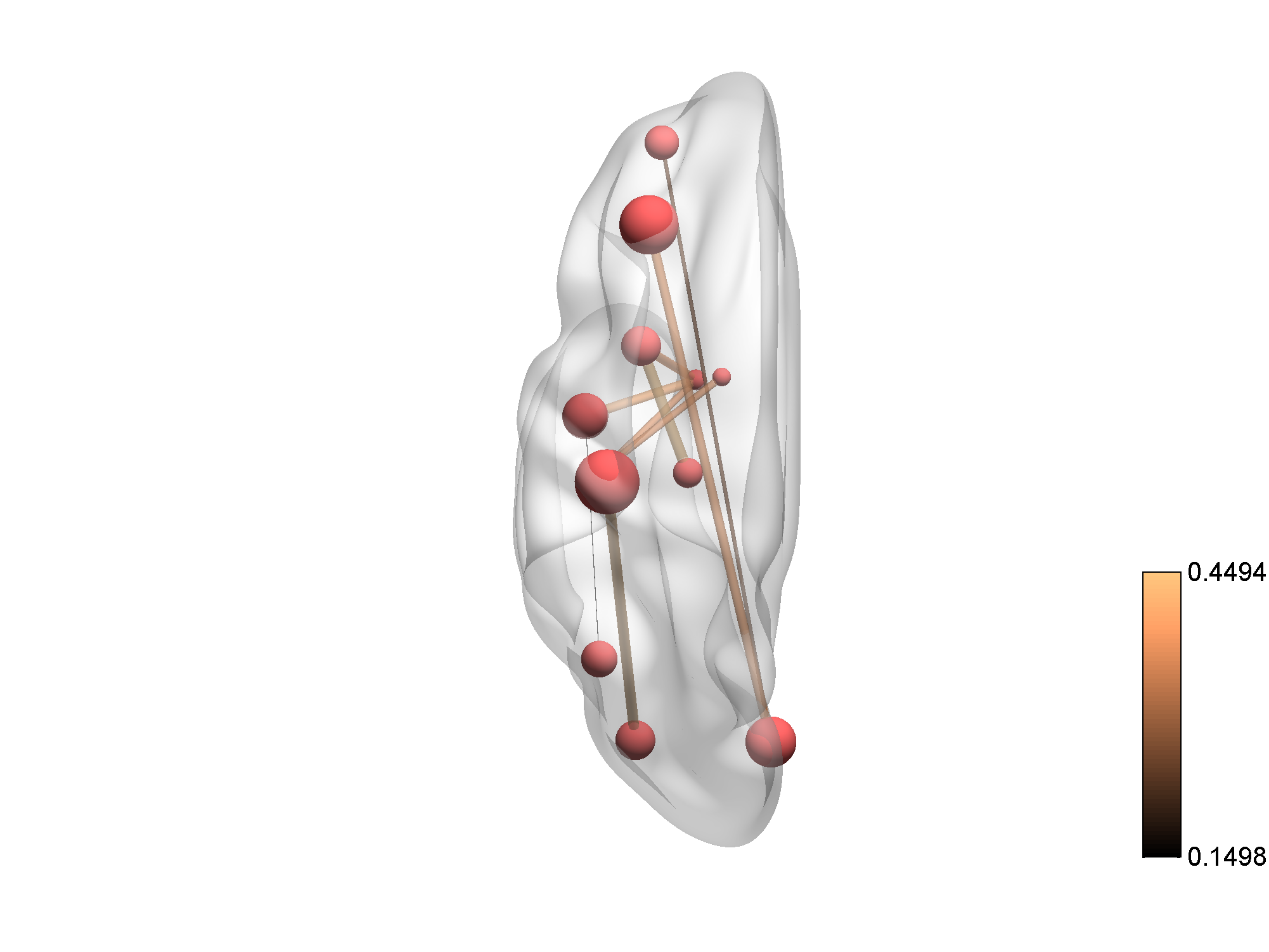


Supplementary Figure E

Right hemisphere：cao > cas 15-28、20-40


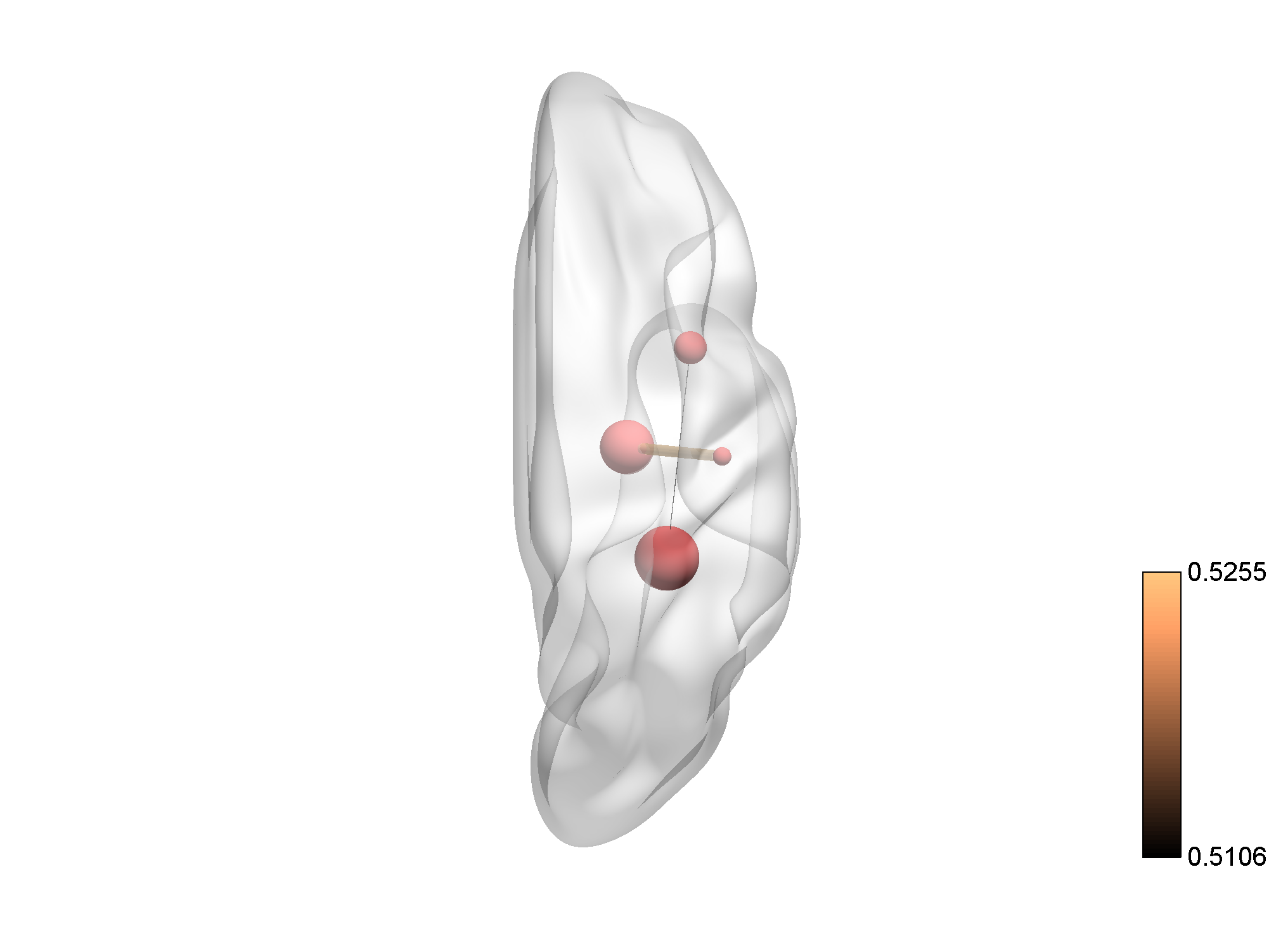


Supplementary Figure F

Right hemisphere：cao> hc 20-40


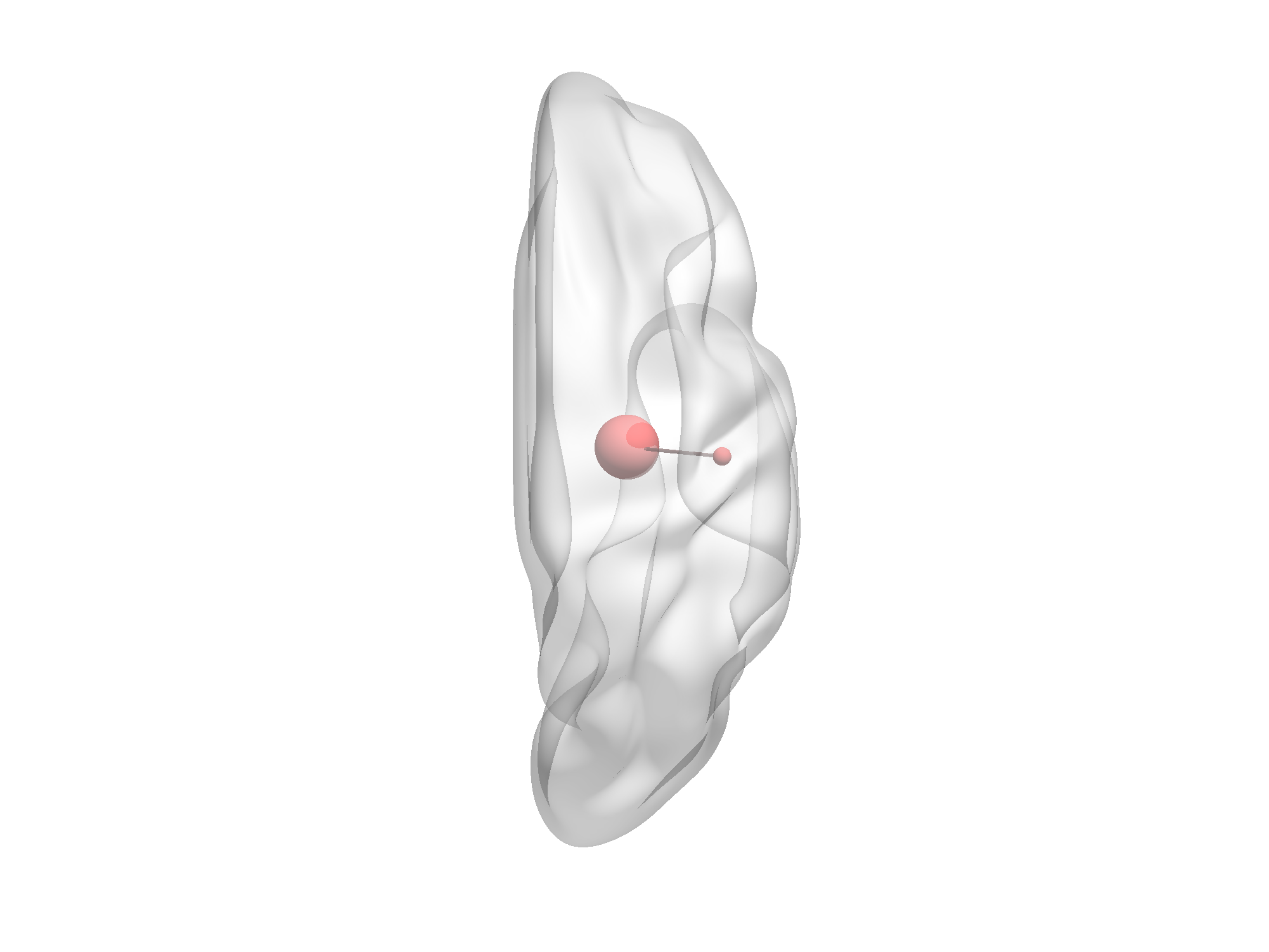


**Additional images of patients with right carotid stenosis and occlusion**Supplementary Figure G

CAO: The right hemisphere < The left hemisphere 9-10、1-15、4-15、9-15、10-15、4-16、7-17、15-17、15-29、17-29、15-30、24-30、28-30、30-34、15-45


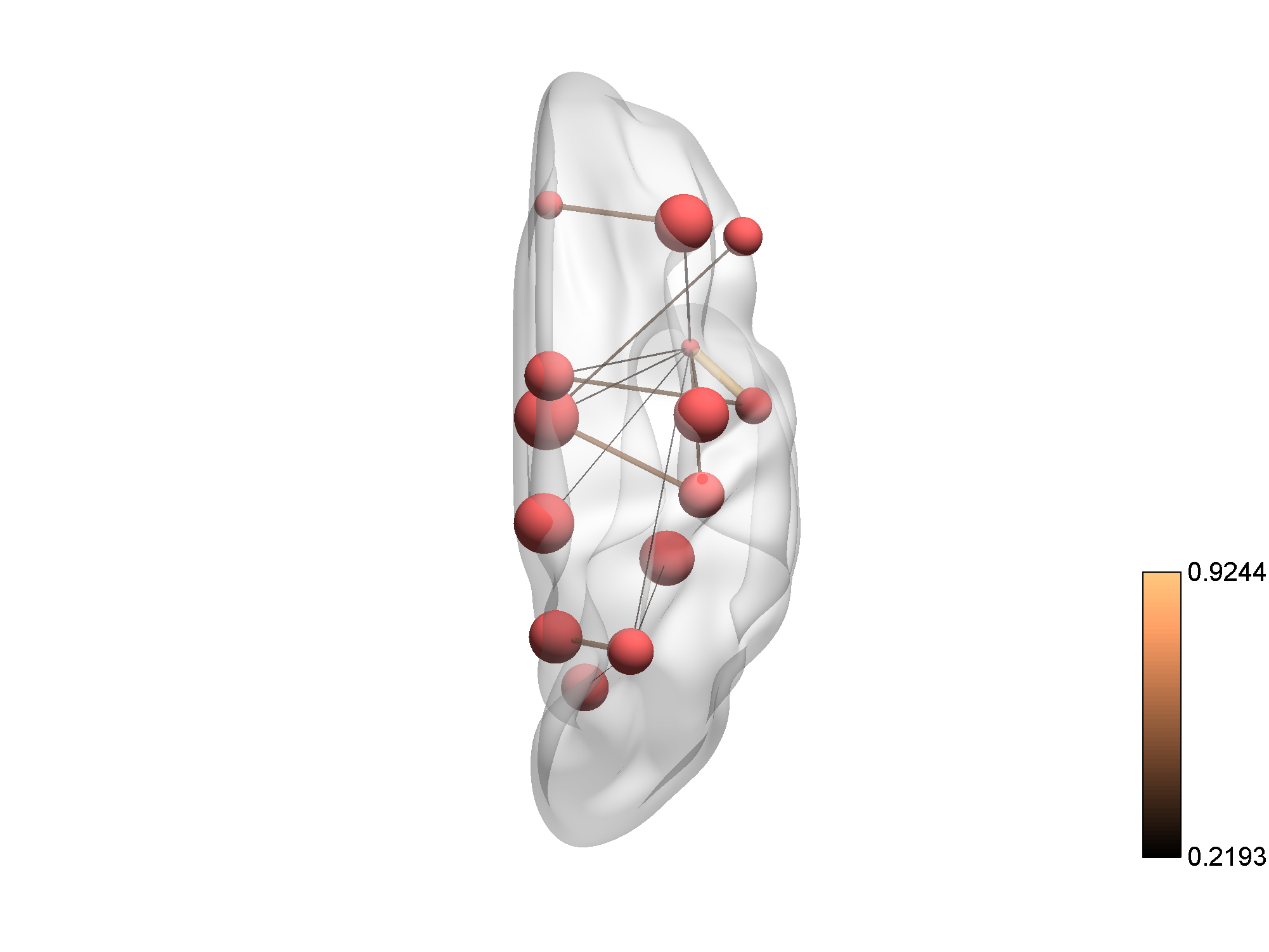


Supplementary Figure H

CAS: The right hemisphere < The left hemisphere 1-15、4-15、9-15、10-15、4-16、7-17、15-17、15-29、17-29、15-30、24-30、28-30、30-34、15-45


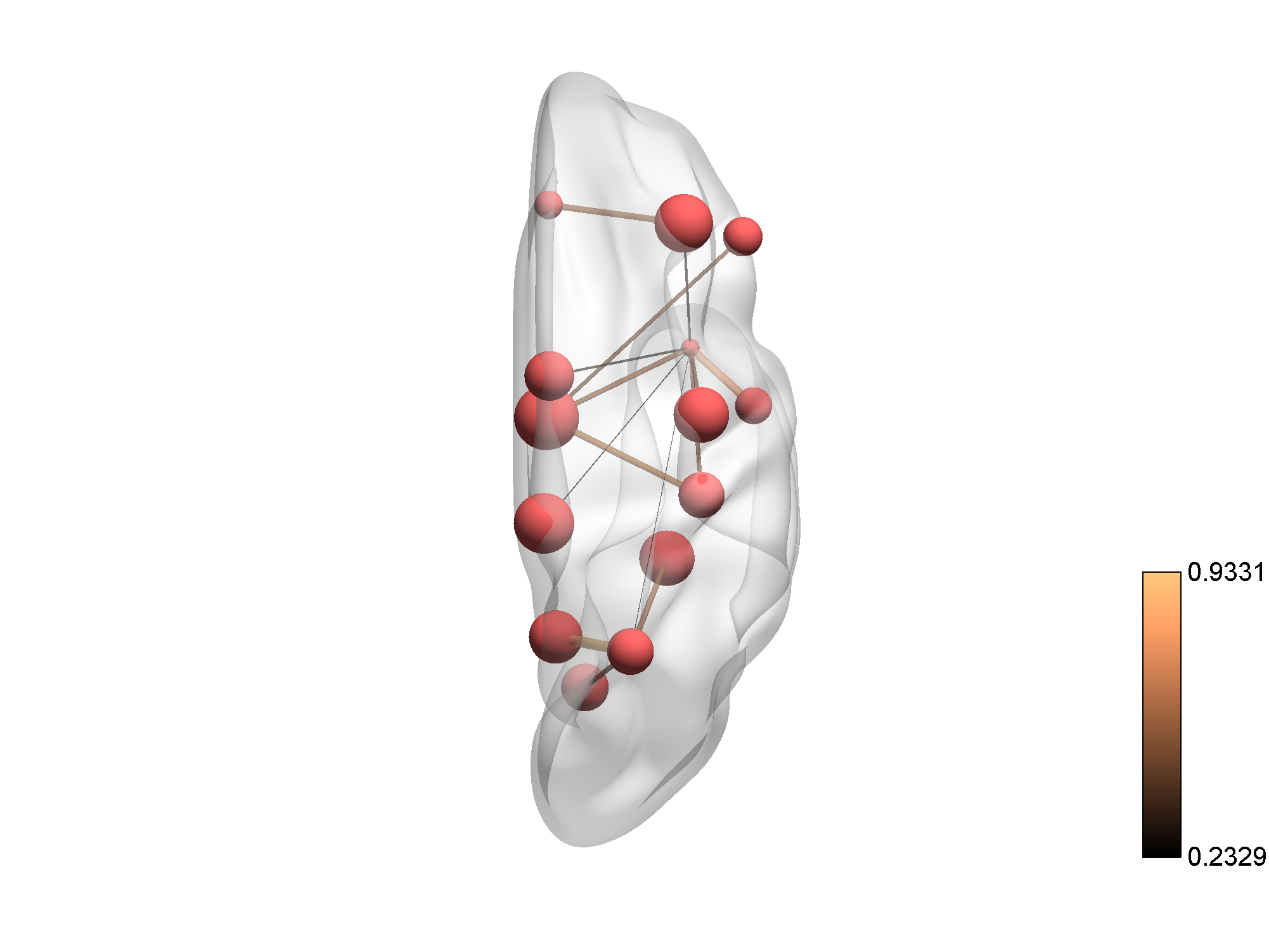


(Attached table: node encoding corresponds to brain region)

| 1 | Precental gyrus | PreCG |
| --- | --- | --- |
| 2 | Superior frontal gyrus, dorsolateral | SFGdor |
| 3 | Superior frontal gyrus, orbital part | ORBsup |
| 4 | Middle frontal gyrus | MFG |
| 5 | Middle frontal gyrus, orbital part | ORBmid |
| 6 | Inferior frontal gyrus, opercular part | IFGoperc |
| 7 | Inferior frontal gyrus, triangular part | IFGtriang |
| 8 | Inferior frontal gyrus, orbital part | ORBinf |
| 9 | Rolandic operculum | ROL |
| 10 | Supplementary motor area | SMA |
| 11 | Olfactory cortex | OLF |
| 12 | Superior frontal gyrus, medial | SFGmed |
| 13 | Superior frontal gyrus, medial orbital | ORBsupmed |
| 14 | Gyrus rectus | REC |
| 15 | Insula | INS |
| 16 | Anterior cingulate and paracingulate gyri | ACG |
| 17 | Median cingulate and paracingulate gyri | DCG |
| 18 | Posterior cingulate gyrus | PCG |
| 19 | Hippocampus | HIP |
| 20 | Parahippocampal gyrus | PHG |
| 21 | Amygdala | AMYG |
| 22 | Calcarine fissure and surrounding cortex | CAL |
| 23 | Cuneus | CUN |
| 24 | Lingual gyrus | LING |
| 25 | Superior occipital gyrus | SOG |
| 26 | Middle occipital gyrus | MOG |
| 27 | Inferior occipital gyrus | IOG |
| 28 | Fusiform gyrus | FFG |
| 29 | Postcentral gyrus | PoCG |
| 30 | Superior parietal gyrus | SPG |
| 31 | Inferior parietal, but supramarginal and angular gyri | IPL |
| 32 | Supramarginal gyrus | SMG |
| 33 | Angular gyrus | ANG |
| 34 | Precuneus | PCUN |
| 35 | Paracentral lobule | PCL |
| 36 | Caudate nucleus | CAU |
| 37 | Lenticular nucleus, putamen | PUT |
| 38 | Lenticular nucleus, pallidum | PAL |
| 39 | Thalamus | THA |
| 40 | Heschl gyrus | HES |
| 41 | Superior temporal gyrus | STG |
| 42 | Temporal pole: superior temporal gyrus | TPOsup |
| 43 | Middle temporal gyrus | MTG |
| 44 | Temporal pole: middle temporal gyrus | TPOmid |
| 45 | Inferior temporal gyrus | ITG |
